# Development of a digitalization concept for assessing patient-reported outcomes in the context of a University Cancer Center – a model for use in clinical routine

**DOI:** 10.1186/s41687-025-00948-4

**Published:** 2025-09-08

**Authors:** Anna Saibold, Michael Koller, Karolina Mueller, Tobias Pukrop, Michael Rechenmacher, Oliver Koelbl, Fabian Pohl, Julia Maurer

**Affiliations:** 1 Department of Information Technology, University Hospital Regensburg, Franz-Josef-Strauss-Allee 11, 93053 Regensburg, Germany; 2 Bavarian Cancer Research Center (BZKF), Regensburg, Germany; 3 Center for Clinical Studies, University Hospital Regensburg, Regensburg, Germany; 4 Department of Internal Medicine 3, University Hospital Regensburg, Regensburg, Germany; 5 Department of Radiation Oncology, University Hospital Regensburg, Regensburg, Germany; 6 University Cancer Center Regensburg, University Hospital Regensburg, Regensburg, Germany

**Keywords:** Patient-reported outcomes, Digitalization concept, Oncological routine screening

## Abstract

**Background:**

Systematic assessment of patient-reported outcomes (PROs) is essential for identifying supportive care needs in oncology. In contrast to paper-based assessments, digital tools offer key advantages such as real-time data availability, improved data quality, and workflow integration. However, digitalization also entails conceptual and technical challenges, including integration into existing clinical systems, data privacy, IT-security, usability, and data quality.

**Digitalization Concept:**

This short report outlines the development and implementation of a modular digitalization strategy for PRO assessment at the University Cancer Center Regensburg. Based on the ONCO-ROUTES screening tool, the digital concept integrates common standards and applies features such as skip logic, metadata tracking, and optional response settings to balance data quality with patient autonomy. PRO data are temporarily stored and retrieved automatically by the tumor documentation system at regular intervals. Patient representatives of the advisory board were involved in the conception from the initial development stages, including focus groups. Particular emphasis was placed on usability and the respective technical setup including necessary communication interfaces. PRO results can be directly integrated into oncological patient records and hospital databases in a structured format and considered in the context of treatment.

**Conclusion:**

The concept and technical solution proved feasible for clinical implementation, enabling structured, patient-centered PRO collection in routine oncology care. Its modular design allows for adaptation to other clinical settings and supports broader efforts to digitally integrate PROs into everyday clinical routine.

## Background

The assessment of patient-reported outcomes (PRO) is essential in oncological trials and is becoming increasingly relevant in routine care. PRO offer valuable insights into the physical, psychological, and social well-being of patients [1, 2]. Structured integration into clinical practice enables patient-centered interventions, thereby improving treatment outcomes and overall quality of life [3, 4].

Regular PRO screenings also allow for continuous symptom monitoring and individualized therapy planning [5, 6]. Additionally, PRO strengthen physician-patient interactions by providing structured data that improve communication and enable informed decision-making [7]. PRO implementation thus contributes to responsive, problem-focused and adaptive care [5].

Digital assessment of PRO offers several advantages, including increased efficiency through reduced documentation workload, as well as improved patient-centered care and data-driven decision-making [8, 9]. Moreover, digital assessments improve data quality, enable direct system integration, and optimize patient monitoring through automated processing and scoring [10, 11].

Despite these benefits, digital PRO assessments are still insufficiently integrated into oncological centers and hospital information systems (HIS). In Germany, this is primarily attributable to a strict interpretation of regulatory frameworks—such as data protection legislation and the EU Medical Devices Regulation [8, 9, 12]—as well as a general hesitancy to embrace digital transformation. Further, technical barriers are insufficient access to devices or internet and usability issues like lengthy questionnaires or non-intuitive interfaces [7,11]. Optimizing usability therefore requires simple design and clear user guidance.

Furthermore, direct access to PRO via HIS is crucial for routine care. All relevant stakeholders—clinicians, care teams, and registries—depend on real-time availability of structured patient data [13, 14]. Interoperability between clinical systems and cancer registries is required to realize the full potential of PRO for healthcare and research [12, 15].

This short report builds on two previously published manuscripts: Maurer et al. [16] outlined the implementation of a structured screening process for patients based on the requirements of oncological treatment, focusing on system integration, data security, and usability [16], while Saibold et al. [17] described the selection and implementation of an oncological database software [17].

The present report provides the background on the development of the PRO digitalization concept at a University Cancer Center based on the established oncological database software, enabling seamless integration of PRO data into the clinical cancer registry. This digitalization concept may serve as a model for other institutions seeking to improve oncological care through sustainable digital PRO assessment in clinical routine.

## Digitalization concept

An interdisciplinary project group was established to ensure the successful development and implementation of a digital PRO assessment system. This group consisted of representatives from various organizational and technical domains, including employees from the software company, IT specialists from the hospital, and the management team of the University Cancer Center. Additionally, the local patient advisory board and clinical experts from different departments within the University Cancer Center were involved to ensure that the digitalization concept aligned with practical clinical needs.

The primary objectives guiding the development of the digitalization concept were: (1) ensuring interoperability between PRO data and patient records in the clinical context, (2) meeting data privacy and IT security standards, and (3) providing user-friendly solutions for both physicians and patients.

The major challenges identified in assessing digital PRO can be broadly categorized into technical and conceptual aspects. Technical challenges include ensuring interoperability within existing data infrastructures, complying with regulatory requirements in Germany, selecting appropriate systems and devices, and especially integrating digital PRO into clinical systems. Conceptual challenges include designing user-friendly interfaces and providing adequate support for patients and healthcare professionals to enhance acceptance and participation. [2–6, 8, 10–12, 15, 18, 19, 7, 20–24]

### Technical aspects

To digitalize PRO assessments, a concept for mobile surveys and screenings was developed at the University Cancer Center. The hospital information system i.s.h.med (Cerner) and the tumor documentation software Onkostar (IT-Choice) were identified as the key systems supporting the digitalization of PRO [17]. Additionally, the commercial PRO module of Onkostar was selected, which runs on a secure server accessible via the internet.

**Fig. 1:**
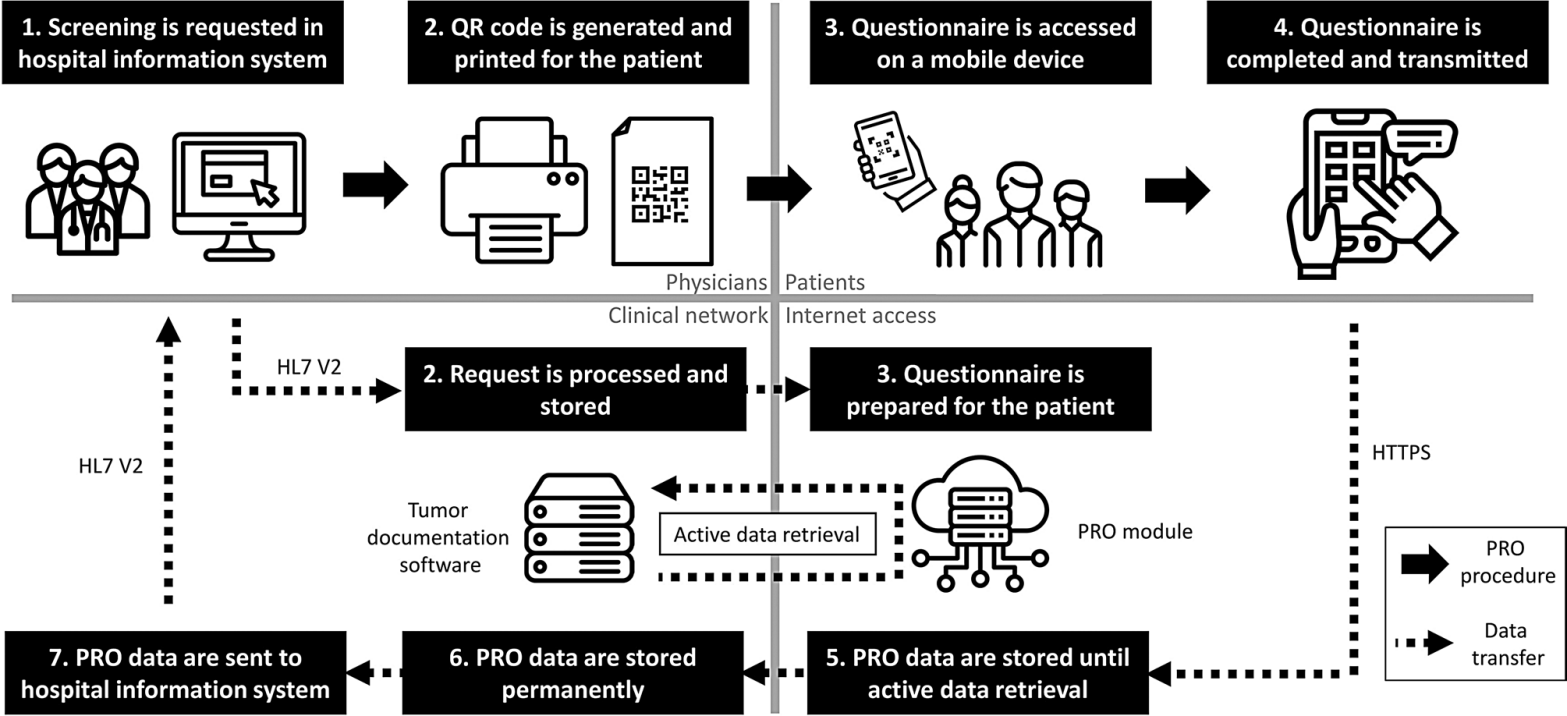
Patient-reported outcome workflow

**Fig. 2:**
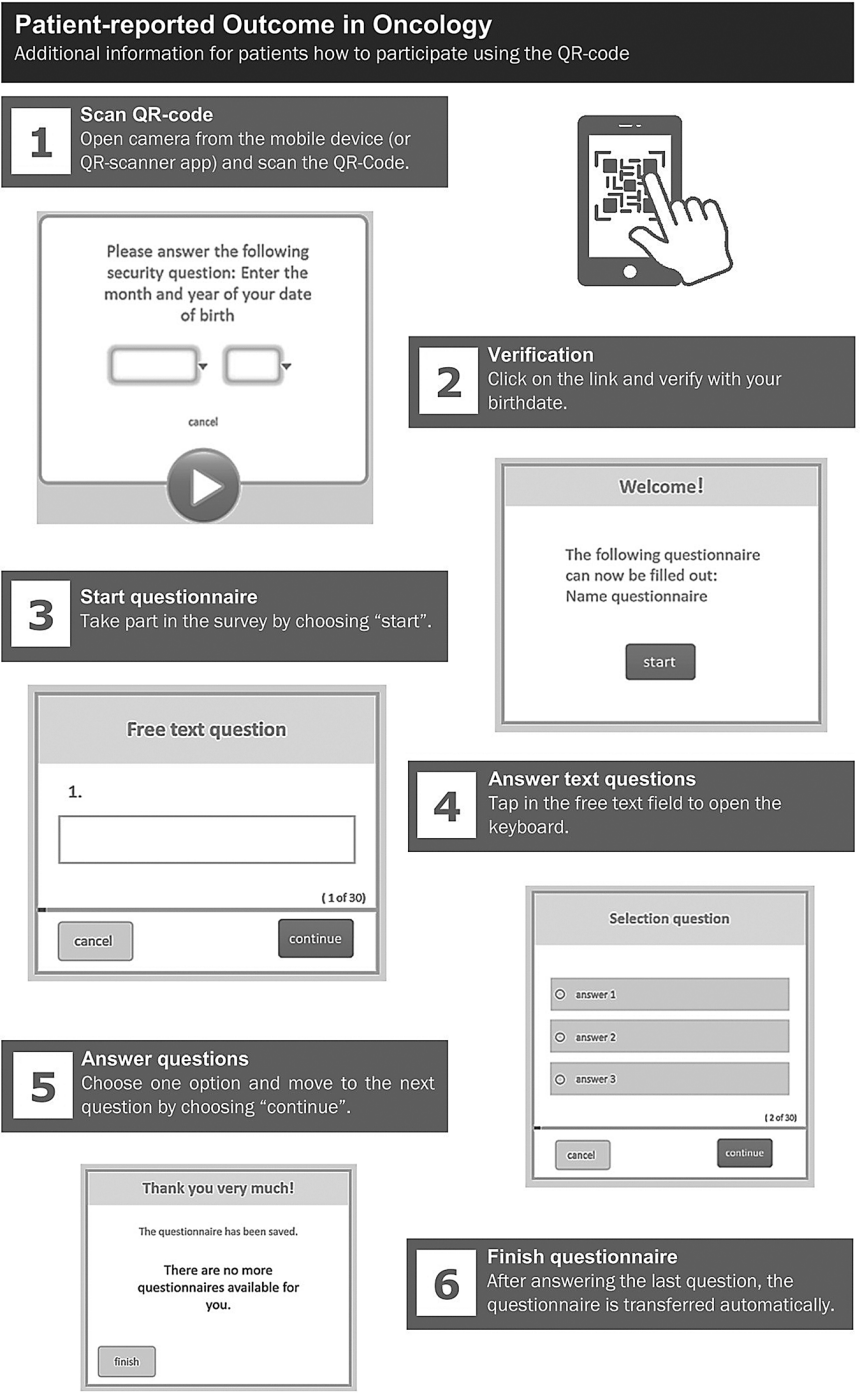
Instruction manual for the digital PRO module

To streamline PRO data collection and integration into oncological records, a workflow was implemented. This novel integration approach was developed to establish standardized communication interfaces, ensuring secure and efficient data exchange between systems. The workflow consists of the following steps (Fig. 1 – steps 1–7):

1. **Screening request in HIS:** The screening is requested by clinical staff.
2. **QR code generation and processing of request:** A QR code that links to a patient-specific digital questionnaire is generated, printed and distributed to the patient along with a detailed instruction manual (Fig. 2). Simultaneously, the request is forwarded via HL7 V2 protocol to the tumor documentation software, in compliance with interoperability standards.
3. **Access via QR code to prepared questionnaire:** The pseudonymized questionnaire is prepared in the PRO module, using an encrypted ID without any patient-identifying data. After scanning the QR code, the patients verify their identity by entering their birth month and year before processing the questionnaire on a mobile device.
4. **Completion and transmission of the questionnaire**: After completion of the questionnaire by the patient, the PRO data are stored in the PRO module.
5. **Temporary storage of data in PRO module:** The PRO module serves as a temporary storage location for newly initialized as well as completed questionnaires prior to automated data retrieval.
6. **Transfer and permanently storage of PRO data:** PRO data are actively retrieved from the PRO module and transferred to the tumor documentation software. This step is performed at regular intervals to retrieve questionnaires stored in the PRO module timely. The data are securely stored linked to the patient record of the cancer registry using the encrypted identifier.
7. **Transfer of PRO data to HIS:** Additionally, the PRO data are automatically sent via HL7 V2 technology to the patients’ record in the HIS. Clinical staff can directly access the results, enabling informed clinical decision-making based on real-time patient-reported data. The assessed PRO data are directly integrated into clinical worklists, this simplifies clinical workflows, such as requesting supportive care services.

The digitalization concept integrates an automated scoring system that enables the derivation of treatment recommendations from PRO data based on predefined trigger responses, as exemplified by ONCO-ROUTES. By evaluating patient-reported symptom burden in real time, the system calculates symptom scores and generates tailored recommendations for supportive therapies in oncological care. These recommendations are derived from defined threshold values and can be individually customized for each questionnaire to accommodate specific clinical needs. The resulting recommendations are then transmitted to the HIS, facilitating automated decision support and optimizing the use of digital PRO data for individualized, patient-centered treatment planning. An interactive overview of completed and pending PRO assessments is accessible to all clinical departments to facilitate efficient monitoring and follow-up.

The key security concept is that only a pseudonymized questionnaire is accessible from the internet, which is not sent but actively collected by the tumor documentations software and matched with the patient data via the encrypted ID.

To further maintain high data security standards and ensure compliance with GDPR and institutional regulations, all servers are locally hosted at the hospital. Stringent security measures ensure that HL7-based data communication is restricted to the internal clinical network, with no external data transfers permitted. To further enhance patient privacy, digital questionnaires are designed to exclude any direct patient identifiers. Additionally, questionnaires are time-limited and expire after a pre-defined period to prevent unauthorized access.

In the event of technical issues, a fallback mechanism is in place, allowing patients to complete paper-based questionnaires with subsequent manual data entry into the system. This method ensures continuity in PRO data collection while minimizing potential disruptions in clinical workflows.

### Conceptual aspects

Utilizing the technical setup, the initially paper-based ONCO-ROUTES questionnaire was transformed into a digital PRO assessment tool. This process included addressing also organizational challenges, ensuring integration into existing clinical workflows, while optimizing usability for both patients and healthcare professionals.

The selection and structure of the ONCO-ROUTES questionnaire is described in detail in the ONCO-ROUTES publication [16]. ONCO-ROUTES was created through guideline review, Delphi rounds, and interdisciplinary focus groups including patient representatives to ensure clinical relevance. Key supportive care domains such as physical functioning, psycho-oncological distress, social needs, and nutrition are covered. Widely accepted instruments like the Distress Thermometer, EORTC QLQ-C15-PAL, and IPOS were prioritized, supplemented by PRO-CTCAE items and self-developed questions.

The digitization of ONCO-ROUTES followed established best-practice principles as outlined by the ISPOR ePRO Task Force. As the modifications applied (e.g., adaptations in navigation, layout, and instructional text) fall into the category of minor changes, equivalence testing was not deemed necessary in accordance with ISPOR guidance. The electronic versions were developed following design best practices for eCOAs, including screen readability, response option accessibility, and platform consistency (smartphone, tablet, browser). [25–27]

The successful implementation of digital questionnaires requires careful consideration of multiple usability factors. To enhance readability and minimize patient fatigue, the digital ONCO-ROUTES questionnaire was designed to display only one question per screen, accompanied by intuitive navigation buttons. This format ensures that users can focus on each question individually, reducing cognitive load and improving response accuracy. For data entry, various input formats were incorporated, including radio buttons, drop-down menus, and free-text fields, depending on the nature of each question. Although making fields mandatory can help ensure data completeness, the project group decided against mandatory responses to prevent meaningless entries and reduce the risk of questionnaire abandonment. Instead, skip logic and branching mechanisms were implemented in selected sections to reduce response burden. Skipped items are systematically documented within the software, allowing differentiation between system-driven skips and patient-initiated omissions. This structured categorization enhances the interpretability and integrity of the dataset.

Given that the PRO module operates via a web-based interface, font sizes dynamically adapt to individual device settings. However, recognizing that smaller screens may impact readability, the system allows users to adjust device or browser settings to enhance visibility.

To provide patients with flexibility and reflect clinical realities (e.g., due to interruptions in treatment or care routines), a “save and return” function was implemented. The expiration period of the questionnaire is manually configurable and was set to a few days. After this period, incomplete surveys are automatically marked as unfinished and excluded from the dataset. While this mechanism introduces a theoretical risk of recall bias—particularly for PRO intended to capture the current symptom burden in a single session—the short time frame and real-time data capture substantially mitigate this concern. Moreover, from a clinical perspective, a change in supportive care needs during few days is usually limited and does not significantly impact the relevance of the responses. Patients are nonetheless encouraged to complete the questionnaire in one sitting whenever possible. Metadata are recorded to track session interruptions, and incomplete or delayed responses are flagged accordingly. This approach enables patient-centered flexibility without compromising data quality or interpretability.

Individual members of the project group, including clinical representatives from various departments of the University Cancer Center, reviewed and approved the digitalization concept on the basis of test data sets in the testing environment of the HIS and Onkostar. Thereby, minor technical issues such as error messages, transfer failures, and layout inconsistencies (e.g., font size adjustments) were identified and refined. Beyond clinical process validation, the concept was reviewed by the local patient advisory board by current and former cancer patients, who were also represented in the initial focus groups, regarding accessibility and usability. During consecutive pilot phases, usability testing was conducted to ensure the systems’ comprehensibility and acceptability; patient feedback was systematically collected and iteratively incorporated to refine the user experience. Results will be reported in future publications

### Lessons learned

One of the major challenges in this project was ensuring secure data transfer between the PRO module, the tumor documentation database, and the HIS while complying with IT security and data privacy regulations under resource constraints. To meet data protection standards, an encrypted identifier was used for pseudonymization. This project successfully accelerated the implementation of digital PRO assessments and achieved seamless integration with existing data systems. The HIS now provides an overview of PRO results, incorporating defined triggers that highlight potential therapy needs. The structured expansion of oncological patient data within the tumor documentation software, combined with cross-departmental HIS connectivity, enhances patient care and supports more efficient clinical decision-making.

A further key challenge was designing a user-friendly interface that minimizes patient burden while ensuring high data completeness. The technical infrastructure is now transferable for routine PRO screenings across oncological departments, improving efficiency and optimizing resource utilization. Additionally, linking PRO data to clinical records enables scientific applications in healthcare research while providing a comprehensive patient overview. In case of ONCO-ROUTES, the technical infrastructure facilitates the identification of supportive care needs and allows therapy adjustments throughout the treatment process. ONCO-ROUTES has thus established a strong foundation for further digital advancements in oncological care, opening new possibilities for personalized and data-driven treatment approaches.

Other challenges related to patient experience and clinical implementation, such as personnel requirements, financial costs, and accessibility issues, are beyond the immediate scope of this work, but will be addressed in future research.

## Conclusion

Our experiences show the feasibility of digital PRO integration and the potential for future technical and conceptual optimizations of the assessment of PRO at the University Cancer Center.

Embedding PRO results into oncological patient records and hospital databases strengthens evidence-based decision-making and expands research opportunities. While initial IT security, data privacy, and usability aspects were challenging, the long-term benefits will outweigh the challenges.

The PRO software setup is highly adaptable and can be integrated into various future projects. The described digitalization concept for PRO assessments may serve as a model for other institutions for assessing PRO in clinical routine.

## Data Availability

The datasets used and/or analyzed during the current study are available from the corresponding author on reasonable request.

## Abbreviations

HIS: Hospital information systems
ONCO-ROUTES: ONCOlogical-ROUTinE-Screening
PRO: Patient-reported outcomes
